# 经支气管超声引导针吸活检在肺癌纵隔分期和胸腔内病变诊断中的应用

**DOI:** 10.3779/j.issn.1009-3419.2010.05.05

**Published:** 2010-05-20

**Authors:** Jens ECKARDT, Peter B. LICHT, 娟 南, 燕 丁

**Affiliations:** 1 Department of Cardiothoracic Surgery, Odense University Hospital, Odense, Denmark; 2 天津医科大学总医院，天津市肺癌研究所，天津市肺癌转移与肿瘤微环境重点实验室

**Keywords:** 气管镜, 胸部肿瘤, 诊断, EBUS, 肺癌

## Abstract

既往研究显示，经支气管超声引导针吸活检（endobronchial ultrasound-guided transbronchial needle aspiration, EBUS-TBNA）是一项微创技术，可用于肺癌患者的纵隔分期、胸腔内病变的诊断、不明原因淋巴结肿大的诊断以及非小细胞肺癌（non-small cell lung cancer, NSCLC）新辅助化疗后的纵隔再分期。

本综述旨在关注EBUS在NSCLC纵隔分期中的作用，以及EBUS在进一步评估通过常规检查仍诊断未明的胸腔内病变中的应用。总之，就NSCLC患者纵隔分期而言，由于EBUS的诊断敏感性和特异性均较高，其为纵隔镜检查的有效替代，亦可用于疑似胸腔病变的活检，且不会给患者带来任何并发症风险。

## 前言

可手术切除的非小细胞肺癌（non-small cell carcinoma, NSCLC）患者常规进行纵隔分期以排除N2疾病。这些患者通常采用作为金标准的纵隔镜检查，然而此项检查也是一项有创外科操作，会给患者带来轻微但明显的风险^[1]^。对于肺癌纵隔分期而言，经支气管超声引导针吸活检（endobronchial ultrasound-guided transbronchial needle aspiration, EBUST B N A）和内镜超声引导针吸活检（endoscopic ultrasound-guided fine needle aspiration, EUS-FNA）是纵隔镜检查的有效替代^[2-6]^。

同样，影像学可疑的诊断未明的胸部病变患者常采用有创检查以确诊。一般情况下，通过常规支气管镜检查或CT引导针吸活检（CT-guided fine needle aspiration, CT-FNA）可确诊，但仍有许多患者诊断不明。本综述旨在关注EBUS在NSCLC纵隔分期中的作用，以及EBUS在进一步评估通过常规检查仍诊断未明的胸腔内病变中的应用。

## 支气管内超声

线性EBUS探头在可弯曲支气管镜的顶端配置有一个凸面的7.5 MHz的传感器，可沿支气管镜的插入方向平行扫描。镜头可弯曲部分的外径为6.7 mm，顶端外径为6.9 mm。视野的角度为80度，视野的方向为前倾35度。通过探头直接接触气管壁或支气管壁或在支气管镜顶端配置一个可注入生理盐水的气囊，可使探头与组织密切接触，这样可以获得超声图像以及随后的超声信号。操作者可以通过两个监视窗同时观察超声图像和支气管镜图像。通过二维图像可测定肿瘤的大小，通过多普勒模式可测定血管结构。通过EBUS探头的工作通道引入一个22 G穿刺针，可实施实时针吸活检。EBUS检查宜在全身麻醉下进行，这样有助于更彻底的检查，清醒患者在中等镇静的情况下亦可行EBUS检查。每一处病变可实施两次或两次以上吸取，以确保有足够的组织用以活检。将吸取的组织置于玻片上并涂片，以便细胞学检查，或置于盐水中制备细胞团，以便组织学检查。如果可实施快速现场细胞学检查，将有助于提高诊断率，但是随着经验的增多，这项检查可以省略。

EBUS的指征有：

●肺癌患者的纵隔分期；

●肺内肿瘤的诊断；

●不明原因的肺门和/或纵隔淋巴结肿大的诊断；

●纵隔肿瘤的诊断；

●NSCLC新辅助化疗后的纵隔再分期。

## 讨论

NSCLC纵隔淋巴结分期的微创检查始于2004年^[6, 7]^。虽然纵隔镜检查仍被认为是纵隔分期的金标准^[8]^，但在某些机构EBUS已经取代了纵隔镜检查^[4, 6, 7]^。尽管比较纵隔镜检查与微创手术的随机研究尚未发表，但是Toloza等撰写的涉及共计5 687例肺癌患者的14项研究的综述发现，标准的经颈纵隔镜检查的敏感性为0.81（范围为0.67-0.92），阴性预测值（negative predictive value, PVneg）为0.91（范围为0.82-0.97）^[9]^。单一EUS检查的敏感性和PVneg分别为0.88和0.77^[9]^，而EBUS与EUS联合检查纵隔淋巴结受累的敏感性高达100%^[10-12]^。这两种检查方法互为补充，均为纵隔检查的可能的微创方法。

单一EBUS检查的总特异性为1.00（95%CI: 0.92-1.00），总敏感性为0.88（95%CI:0.79-0.94）^[13]^。因为EUS与EBUS联合检查对敏感性和特异性的提高甚微，故所有患者均采用这种联合检查是否有益尚存争议。

经由支气管镜检查和/或CT引导活检等常规诊断方法后诊断未明的影像学可疑肺实质肿瘤、纵隔淋巴结肿大或纵隔肿瘤的患者对临床医生提出了挑战。

支气管镜检查的诊断率为20%-60%^[14, 15]^。CT-FNA检查周围型肿瘤的总敏感性为90%^[15]^，检测纵隔肿瘤的总敏感性为83%^[16]^，探查直径小于或大于2 cm的周围型肿瘤的敏感性分别为0.34和0.63。

这些患者通常需进行创伤较大的检查，如纵隔镜检查、胸腔镜检查甚至开胸手术，但是这些有创检查均会给患者带来轻微但明确的风险，而且价格昂贵。EBUS是一种相对较新的诊断方法，创伤较小且性价比较高，因为其可门诊实施。

我们已证实在常规实施支气管镜检查或CF-FNA后仍诊断未明的患者中，大约45%的患者通过微创EBUS可确诊^[18]^。这一诊断率低于通常报道的已知肺癌患者纵隔分期的诊断率^[2, 6]^，但是区别这两类患者人群尤为重要。EBUS作为早期诊断方法，有可能提高确诊率。其他研究者采用EBUS作为提高中央型疑似肿瘤的早期诊断方法，诊断成功率较高^[19]^。另一项研究指出，就小于3 cm的周围型肺肿瘤而言，作为早期诊断方法，EBUS优于经支气管活检^[30]^。

EBUS和其它方法的诊断率均有赖于肿瘤部位，而且对中央型肿瘤的诊断率高于周围型肿瘤^[15]^。我们发现，60%的中央型肿瘤患者与仅32%的周围型肿瘤患者可被确诊^[18]^。

其他研究者认为，EBUS可用于NSCLC患者新辅助化疗后的再分期，研究表明其敏感性为76%，特异性为100%，诊断准确率为77%，但是由于PVneg较低（20%），所以阴性结果应在开胸手术前经外科分期得到证实^[2, 21]^。如果需要，EBUS可以常规再次实施，而且不会使患者的风险增加。EBUS是一种安全的方法，且未见有严重并发症的报道^[3, 5]^，而再次实施纵隔镜检查的情况则不甚相同。

一项有趣的研究关注了EBUS的学习曲线，并表明学习这种方法所需的次数仅为10次^[22]^。因此，在大多数机构中，从创伤较大的纵隔镜检查过度到EBUS微创方法均比较容易。

总之，就NSCLC患者纵隔分期而言，由于EBUS的敏感性和特异性均较高，故其为纵隔镜检查的有效替代，亦可用于各种疑似胸部病变的诊断。EUS联合EBUS较单一EBUS的敏感性和特异性更高。对于诊断未明的胸腔内病变患者，EBUS的确诊率为50%，而且不会给患者带来并发症风险。EBUS的学习曲线为10次。
